# The cell-type specific transcriptome in human adipose tissue and influence of obesity on adipocyte progenitors

**DOI:** 10.1038/sdata.2017.164

**Published:** 2017-10-31

**Authors:** Anna Ehrlund, Juan R. Acosta, Christel Björk, Per Hedén, Iyadh Douagi, Peter Arner, Jurga Laurencikiene

**Affiliations:** 1 Lipid Laboratory, Department of Medicine Huddinge, Karolinska Institutet, Stockholm SE-14186, Sweden; 2 Akademikliniken, Storängsvägen 10, Stockholm SE-115 42, Sweden; 3 Center for Hematology and Regenerative Medicine (HERM), Department of Medicine Huddinge, Karolinska Institutet, Stockholm SE-14186, Sweden

**Keywords:** Obesity, Type 2 diabetes, Microarray analysis, Adipocytes

## Abstract

Obesity affects gene expression and metabolism of white adipose tissue (WAT), which results in insulin resistance (IR) and type 2 diabetes. However, WAT is a heterogeneous organ containing many cell types that might respond differently to obesity-induced changes. We performed flow cytometry sorting and RNA expression profiling by microarray of major WAT cell types (adipocytes, CD45−/CD31−/CD34+ progenitors, CD45+/CD14+ monocytes/ macrophages, CD45+/CD14− leukocytes), which allowed us to identify genes enriched in specific cell fractions. Additionally, we included adipocytes and adipocyte progenitor cells obtained from lean and obese individuals. Taken together, we provide a detailed gene expression atlas of major human adipose tissue resident cell types for clinical/basic research and using this dataset provide lists of cell-type specific genes that are of interest for metabolic research.

## Background and Summary

Obesity and its comorbidities are major health problems in modern society. Expansion of white adipose tissue (WAT) is often associated with fat cell hypertrophy (few, but large cells), low-grade chronic inflammation, changes in WAT-resident immune cell populations and altered secretion of proteins (adipokines) and lipids, all of which are important for the development of insulin resistance (IR), type 2 diabetes, hyperlipidemia and atherosclerosis^1–3^.

WAT is a heterogeneous organ composed of many cell types including adipocytes, adipocyte progenitor cells, endothelial cells, fibroblasts and various types of immune cells^4–6^. Less than 50% of the cells are the tissue specific fat cells. Obesity has a complex impact on WAT by affecting distinct cell populations differently. In obese WAT, macrophages acquire a pro-inflammatory phenotype^7^, the relative abundance of lymphocyte populations is changed^8^, and endothelial cells are activated^9^. In addition, WAT expansion causes changes in the progenitor cell population, and a change of caloric intake can affect differentiation/recruitment of new fat cells^10,11^.

WAT metabolic alterations caused by obesity are also reflected in gene expression, a measurement often used in clinical studies. mRNA profiling of WAT obtained from lean/obese individuals or before/after weight reduction is commonplace, e.g. refs 12–15. However, such an approach neither gives insights into the changes within specific cell types nor enables prediction of intracellular gene regulatory networks as obesity-affected genes might be differentially expressed in various WAT cell types. Only a few studies have tried to address these questions comparing gene expression in paired samples of SVF and adipocytes^16^ or analyzing expression in one particular cell fraction (magnetic-bead sorted macrophages/monocytes)^17^. Systemic comparison of transcriptomes in WAT cell types is lacking, but is of great interest for the field.

The aims of the current study were to determine transcriptomic profiles of the major cell types in human WAT and thus enable investigations of previously published obesity-regulated genes^13^, or other WAT-genes of interest, in relevant WAT cell populations. We also aimed to investigate how obesity affects gene expression and function of human adipocyte progenitor cells. The adipocyte progenitors are highly relevant for WAT morphology and metabolic phenotype as hyperplastic WAT (many, small fat cells) reflects efficient recruitment/differentiation of adipocyte progenitors and is associated with a favourable metabolic profile while hypertrophic WAT (few, large fat cells) is closely linked to a pernicious metabolic profile and IR^18,19^. The adipocytes have direct effects on whole body energy homeostasis by regulating lipid turnover and secretion of adipokines like adiponectin and leptin.

**Figure 1:**
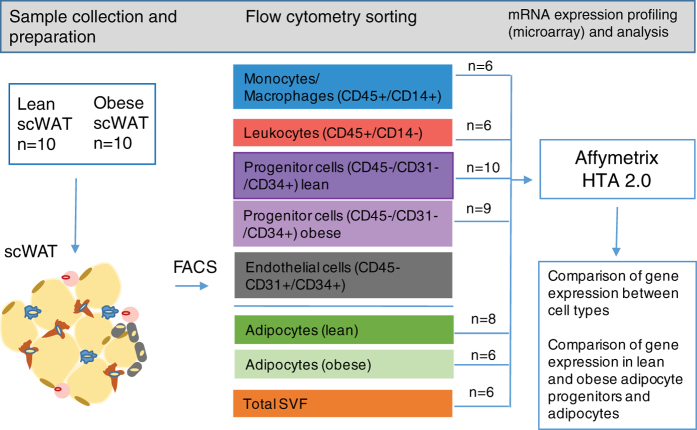
Outline of the project.

To address these questions in human WAT, we used flow cytometry sorting of the stroma vascular fraction (SVF) of WAT, performed transcriptional profiling in four major cell populations, and compared mRNA expression in adipocyte progenitor cells and adipocytes from lean and obese individuals (Fig. 1). Here we provide a full transcriptomic dataset for major WAT cell types as well as trancriptome of adipocyte progenitors and adipocytes obtained from lean and obese individuals. Analysis of fraction-enriched genes is also provided as an additional useful tool for the researchers in the field.

## Methods

### Human subjects and metabolic measurements

Subcutaneous (sc)WAT from 10 healthy obese and 10 non-obese healthy individuals undergoing cosmetic plastic surgery was collected (Table 1). Obesity-regulated genes have been defined in WAT from 56 non-obese/obese women^13^. All subjects were given written and oral information about the study before they provided their written informed consent. The study was approved by the regional committee on ethics at Karolinska Institutet.

### Flow cytometry

scWAT SVF was isolated and cryopreserved, then stained and analyzed/collected by FACS analysis as described^20^. Mature adipocytes were prepared in parallel of SVF preparation as described^21^. The antibodies used are specified in Table 2. Progenitor cells (CD45−/CD34+/CD31−), endothelial cells (CD45−/CD34+/CD31+), monocytes/macrophages (CD45+/CD14+) and leukocytes (CD45+/CD14−) were collected for RNA purification. In addition, the occurrence of T-cell population (CD45+/CD3+/CD14−) was recorded.

### RNA extraction

RNA from FACS-sorted cell fractions and SVF were extracted with RNeasy Micro Kit (Qiagen, Hilden, Germany) and from adipocytes with RNeasy Lipid tissue kit (Qiagen) in accordance with the manufacturer’s recommendations.

### Microarray analysis

RNA expression was analyzed on Affymetrix GeneChip Human Transcriptome Array 2.0 (Affymetrix Inc., Santa Clara, CA) in accordance with the manufacturer’s instructions. Arrays were normalized (RMA, transcript cluster level) in the Expression Console (Affymetrix, Thermo Scientific).

### Statistical analysis of microarray data

After RMA normalization in Affymetrix Expression console software all further analysis of microarray data was carried out in R statistical software (http://CRAN.R-project.org/).

#### Quality control

After RMA normalization, the array quality was assessed using the ArrayQualityMetrics package in R^22^.

#### Enrichment in adipocyte, adipocyte progenitor, macrophage and leukocyte fractions

We excluded array control transcripts before further analysis (by selecting only transcripts with affymetrix category ‘main’) from this step onward. Our data set contains adipocyte, adipocyte progenitor, macrophage and leukocyte microarrays from RNA from 6 non-obese female subjects. To identify genes enriched in one of the fractions versus all others we used the Bioconductor R-package LIMMA^23^. We made paired, pairwise comparisons of all fractions and selected probes for each fraction that had significantly (Benjamini-Hochberg corrected *P*-value<0.05) higher expression levels in that fraction compared to all others. We also calculated moderated F-statistics for all genes and filtered out any genes that were not significant (Benjamini-Hochberg adjusted *P*-value<0.05) according to that analysis. The R-code for this analysis can be found in Data Citation 1.

### Differential expression in progenitors and adipocytes

Differential gene expression in progenitors and adipocytes from non-obese and obese patients was also determined using LIMMA^23^. Array probes without gene symbol annotation were filtered out before LIMMA analysis.

## Data Records

All microarray data are accessible on GEO (GSE80654) (Data Citation 2) and analysis files are provided as supplemental tables (Data Citation 3, Data Citation 4, Data Citation 5 and Data Citation 6).

### Description of files

File 1: Microarray data are decribed in Data Citation 2.

File 2: Tables of fraction-specific genes (Data Citation 3). RNA from non-obese paired adipocyte, leukocyte, macrophage/monocyte and adipocyte progenitor fractions (from six non-obese women) were analyzed and cell-fraction enriched genes were defined as described under methods and R-code (Data Citation 1 and Data Citation 3).

File 3: Pairwise comparison of adipose cell fractions: RNA from non-obese paired adipocyte, leukocyte, macrophage/monocyte and adipocyte progenitor were compared to each other and differentially expressed genes defined as described in methods and R-code (Data Citation 4).

File 4: Table with obesity-regulated genes in progenitors. Effect of obesity on RNA expression in adipocyte progenitor cells was analyzed using samples from 10 non-obese women, 7 obese women and 2 obese men (Data Citation 5).

File 5: Table with obesity-regulated genes in adipocytes. Effect of obesity on RNA expression in purified mature adipocytes was analyzed using samples from 8 lean women, 5 obese women and 1 obese man (Data Citation 6).

## Technical Validation

### Quality control of FACS sorting

**Figure 2:**
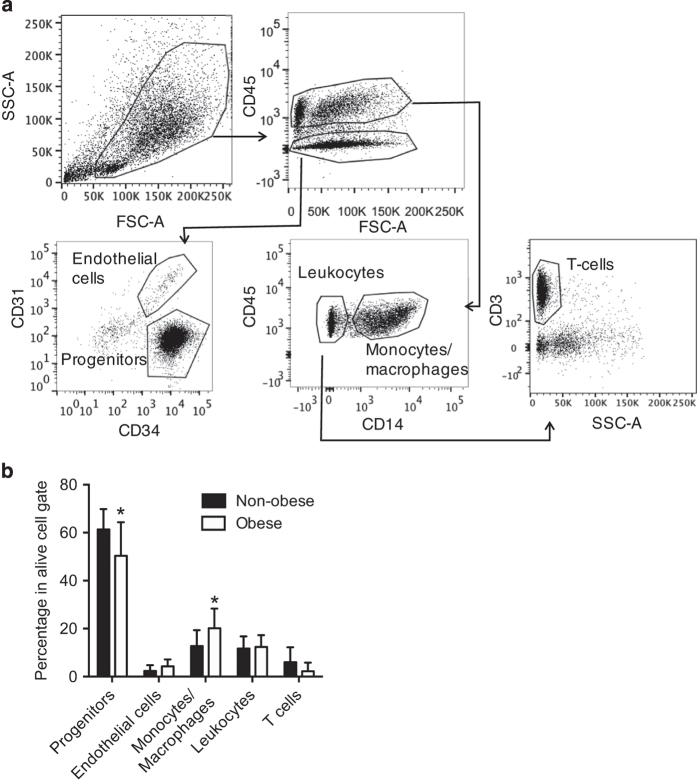
Flow cytometry analysis of human subcutaneous adipose tissue stroma vascular fraction (SVF). (**a**) Representative FACS profiles and gating strategy with marked populations of cells used for analysis. (**b**) Relative frequency of cell populations in lean and obese SVF (*P<0.05, lean *n*=10, obese *n*=9). Non-obese and obese groups were compared by multiple *T*-test. Means and standard deviations are shown.

**Figure 3:**
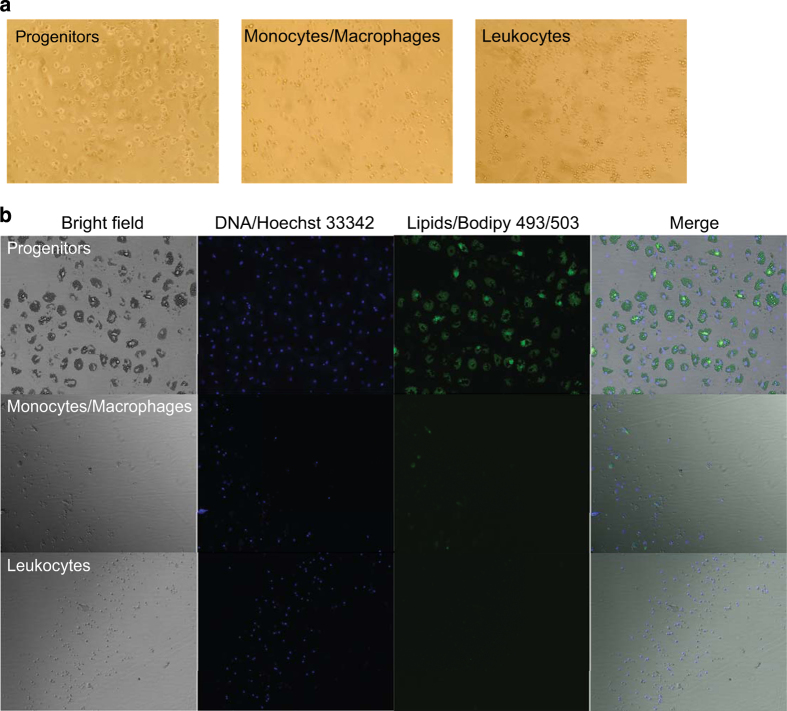
Differentiation of FACS-sorted fractions. (**a**) Pictures of the cells one day after FACS-sorting/plating, before the induction of adipogenic differentiation. (**b**) Pictures of the cells at day 13 of differentiation. DNA dye Hoechst was used to visualize nuclei representing total amount of cells and neutral lipid dye Bodipy 493/503 was used to visualize lipid droplets.

Purity of each FACS-sorted fraction was evaluated by post-sort analysis. Mean purity of adipocyte progenitor cells was 97.7±1.68%; endothelial cells: 77.5±11.9%; monocytes/macrophages: 95.1±2.9%; leukocytes: 98.8±0.87%. Individual values of purity for each sample are shown in Table 1. Viability of cells was generally between 85% and 70% and was determined by 7-aminoactinomycin D (7-AAD)^20^ and by distribution on SSC/FSC scatters where alive and dead cells constituted distinct populations. Samples with lower viability than 65% were not used for the analysis. SVF viability for each sample is indicated in Table 1. Functional validation of FACS sorting quality was performed by inducing adipogenesis *in vitro* in all sorted fractions. Only progenitor cells and total SVF had capacity to differentiate. Purity of adipocyte preparation was examined in an earlier study and was found to be 99%^24^. A sorting scheme, flow cytometry plots, gating strategy and cell fraction frequencies are shown in Fig. 2. Differentiation ability of progenitor, monocyte/macrophage and leukocyte fractions are shown in Fig. 3.

### Quality control of RNA integrity

To determine RNA quality, Agilent 2100 Bioanalyzer (Agilent Technologies Inc., Santa Clara, CA) was used. The integrity of RNA was calculated using RIN (RNA integrity number) algorithm, where higher numbers indicate higher quality, a maximum value being 10. Mean RIN value of the samples was 7.9 and lowest acceptable RIN in this study was 6.6. In adipocyte fraction, RIN values reflected well the amount of viable cells in the preparation.

### Quality control of microarray profiling

**Figure 4:**
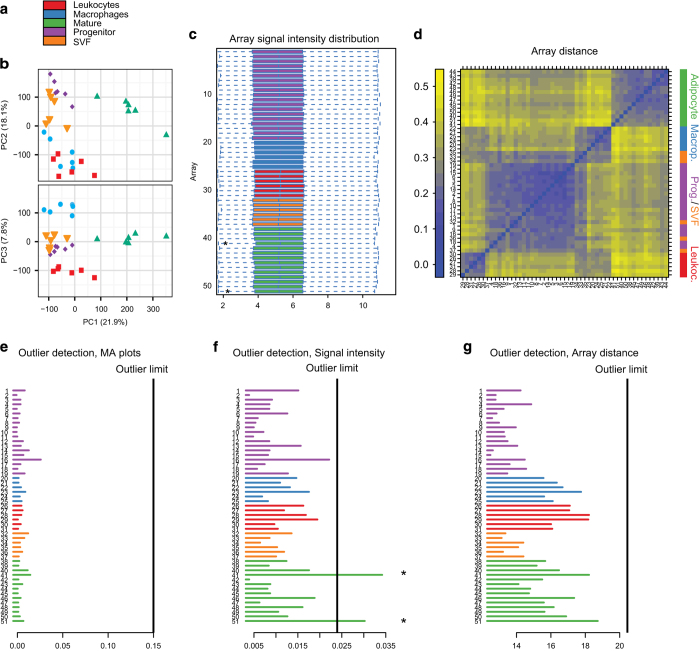
Quality control of microarray data. (**a**) Color coding of samples throughout this figure. (**b**) Principal component analysis plot with PC1 versus PC2 and PC1 versus PC3 showing separation of the sorted fractions. (**c**) Box plot of array distance distributions after RMA normalization. (**d**) Heatmap of distance between arrays. Color scale covers the range of distances encountered between the arrays. (**e**,**f**,**g**) Outlier detection in array QualityMetrics based on the specified parameters. Only two possible outliers were detected, one adipocyte non-obese and one adipocyte obese sample (* in **f**). As this was based on signal intensity distribution alone and none of the other two outlier detection method called these (or other) samples, we decided to keep them in the analysis.

**Figure 5:**
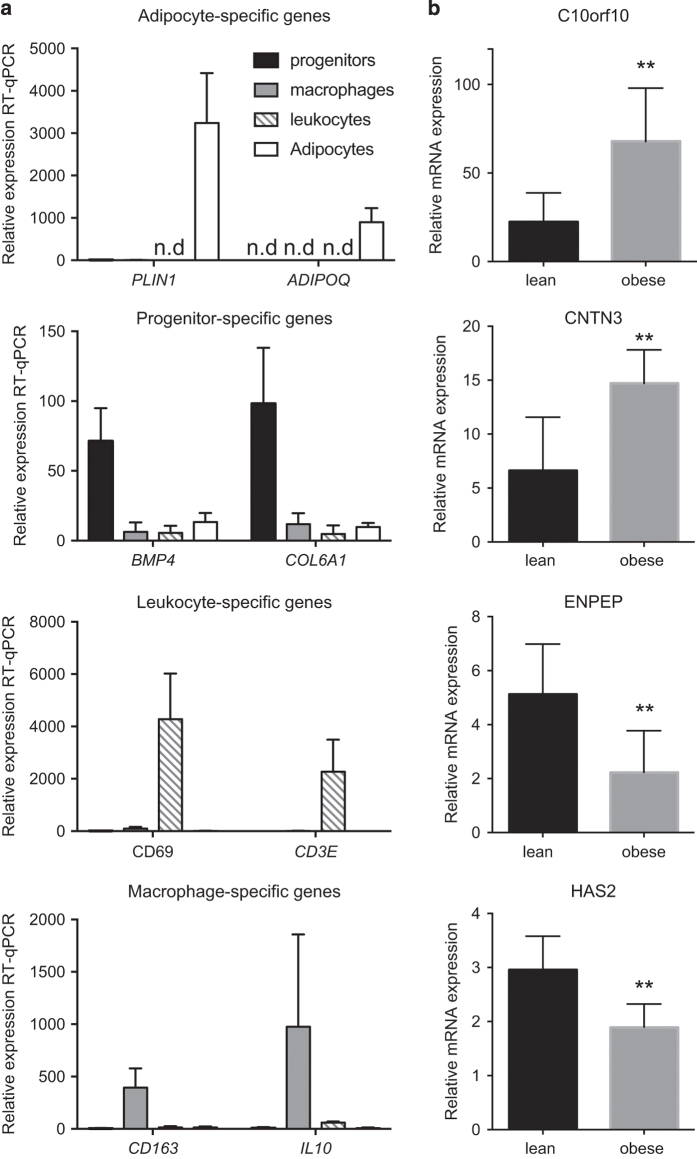
Technical validation of microarray by RT-qPCR. (**a**) Relative expression of selected known fraction-specific genes in four cell fractions from lean individuals (*n*=3–5). Groups are compared by multiple *T*-test and all fraction specific genes are significantly higher in the expected fraction (adjusted *P*<0.05). Means and standard deviations are shown. (**b**) Relative expression of selected genes in lean (*n*=8) and obese (*n*=7) women. Inclusion of 2 obese men in the obese group (original microarray cohort) does not affect significance for any of the tested genes. Groups are compared by *T*-test (**P<0.01). Mean and standard deviations are shown. n.d.—not detected.

RMA normalized data was quality controlled using the ArrayQualityMetrics package^22^ in R (Fig. 4c–g). Principal component analysis performed in R showed that samples grouped on cell fraction. The two immune cell fractions, leukocytes and macrophages/monocytes were separated by PC1/PC3 (Fig. 4b) and formed distinct clusters in array distance distribution pseudo-heat map (Fig. 4d). The array signal intensity indicated that two adipocyte samples (JL41_M_4 (non-obese) and JL51_M_17 (obese)) as outliers (Fig. 4c,f) but as neither MA-plots nor array distance analysis identified these samples as outliers (Fig. 4e,g), nor did the PCA analysis show these samples to be distant from others. Thus, we kept them in our downstream analyses. Quality of microarray profiling was also verified by RT-qPCR examining expression of known cell-type-enriched genes in all four major SVF fractions (Fig. 5a) and obesity-regulation of genes in progenitor cell fraction (Fig. 5b) (primers listed in Table 3).

## Usage Notes

### Genes enriched in specific WAT cell fractions

**Figure 6:**
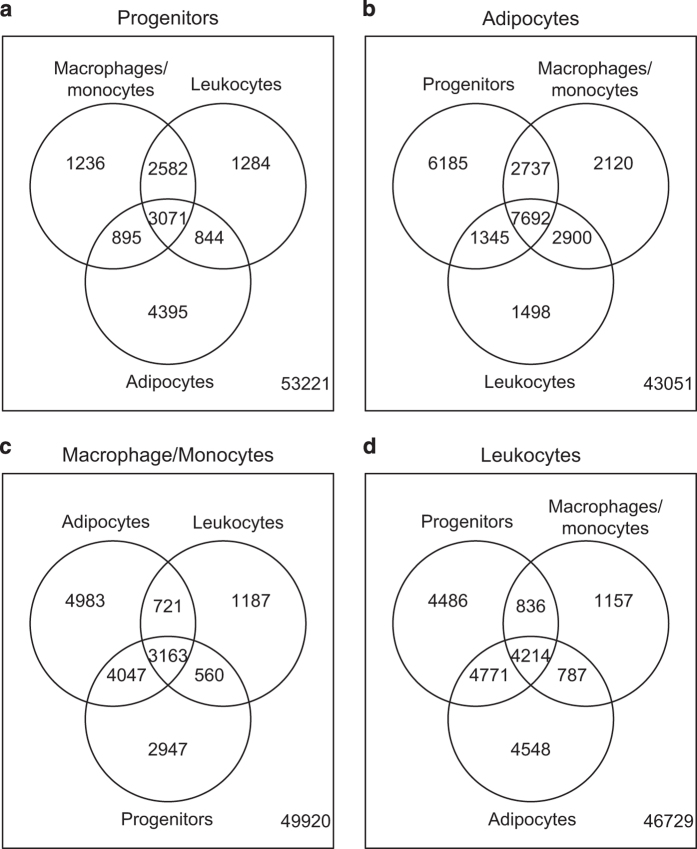
Venn Diagrams of differentially expressed genes compared to other cell fractions. Genes differentially expressed in adipocyte progenitors (**a**), adipocytes (**b**), macrophages/monocytes (**c**), leukocytes (**d**). Amount of genes enriched in the indicated fraction compared to the other three is shown in the middle of the graphs.

In clinical studies, it is often impossible for ethical reasons to obtain enough material to study the effect of different conditions/treatments in the individual cell types of WAT. However, when moving from gene associations to functional studies, the cell type that a gene is primarily expressed in is a crucial clue for designing experiments. Our data set provides a way to assess this, and we provide lists of genes enriched in each of the four cell types we sorted (Data Citation 3). Besides fraction-enriched gene lists, tables include mean expression of the gene in a fraction where it is enriched, log fold change (logFC) compared to each of other fractions and adjusted *P* values (adj.P.Val). Minimal logFC and maximal adj.P.Val against other fractions are included as separate columns to enable easy sorting of the data. A table where 100 highest-ranked genes from each fraction (based on highest logFC_min and lowest adj.P.val_max) is also provided (Data Citation 3). We also provide pairwise comparisons between all fractions so that researchers can quickly check the magnitude of the differential expression for a specific gene (Data Citation 4). The results are summarized in Venn diagrams (Fig. 6a–d).

Our enrichment analysis is well in line with previously reported data. For example the well known ‘markers’ Adiponectin (ADIPOQ), Leptin (LEP) and Perilipin-1 (PLIN1) were among the top enriched adipocyte genes, CD3G and CD69 were enriched in leukocytes, MMP2 and COL1A2-in adipocyte progenitors. In the monocyte/macrophage fraction we found 23 out of 24 earlier reported WAT macrophage-specific genes^17^ among the most enriched. Only HLA-DRA from the previous study was not defined as macrophage/monocyte-enriched, which goes well with it’s reported expression in all types of antigen-presenting cells, such as B-lympocytes, dendritic cells and others^25^. There are also lesser known fraction-enriched genes, of particular interest may be the non-coding genes, that to date have not been well characterized.

### Splicing and non-coding transcripts

The Human transcriptome 2.0 arrays contain exon level information and can be used to analyze splicing using e.g., the affymetrix software ‘Transcriptome analysis console’ that is available for free download on Affymetrix/ThermoFisher Scientific’s webpage https://www.thermofisher.com/se/en/home/life-science/microarray-analysis/microarray-data-analysis/genechip-array-annotation-files.html. This analysis can be useful for determining e.g., differential splicing between cell types, or the expression of a specific splice variant in a cell type.

Furthermore, the HTA2.0 array contains probes for many non-protein coding transcripts, which many other older arrays do not. Thus, this data set can be of specific importance for researchers in e.g., the lncRNA field. Annotation to all included probes can be obtained from Affymetrix/Thermo Scientific’s webpage as indicated above.

### Effects of obesity on scWAT adipocyte progenitor cells

To investigate how gene expression in human adipose progenitors is affected by obesity, we performed microarray analysis on this cell fraction in 10 non-obese and 9 obese individuals. We were primarily interested in annotated genes so we filtered out all probesets without an associated gene symbol before the start of the analysis. When global gene expression in non-obese and obese WAT progenitors was compared, all multiple hypothesis corrected *P*-values were >0.05, probably due to small cohort size and the still large amount of transcripts tested. However, even if false discovery rate is rather high in this data set alone, it may still be used for hypothesis generation, especially when combined with other data and perhaps also with cut-offs on e.g., gene expression fold change. To see whether such an approach had any merit we selected genes that had an unadjusted *P*-value <0.05 and ≥50% up-/downregulation. This gave 35 genes (Data Citation 5), including DKK1^26^, CD36^27^ and BMP2^28^, known to be involved in regulation of adipogenesis and progenitor proliferation. To add more value to biological significance of our findings, we have selected genes that are regulated by obesity in progenitors and highly enriched in this fraction (15 out of 34 genes). We assumed that in intact adipose tissue, most of the expression detected from these genes is coming from progenitor cells. Therefore, we investigated obesity-regulation of these 15 genes in the earlier published cohort reporting obesity-regulated genes in intact WAT^13^. Out of 15 genes, 13 were found in this dataset and 9 of them were regulated by obesity with 5% FDR. This suggests that even this small dataset of obesity regulated-progenitor genes can be used for hypothesis generation before deeper functional studies when combined with other data sets or in larger transcriptomic studies.

## Additional information

**How to cite this article:** Ehrlund, A. *et al.* The cell-type specific transcriptome in human adipose tissue and influence of obesity on adipocyte progenitors. *Sci. Data* 4:170164 doi: 10.1038/sdata.2017.164 (2017).

**Publisher’s note:** Springer Nature remains neutral with regard to jurisdictional claims in published maps and institutional affiliations.

## Supplementary Material



## Figures and Tables

**Table 1:** Patient characteristics and purity of sorted cell fractions*.

**Patient ID**	**Patient group**	**BMI**	**Age**	**Gender**	**Purity of sorted populations (%)**	**Viability of SVF (%)**^**#**^		
					**Progenitors**		**Macrophages**	**Leukocytes**
142-2	lean	25	38	F	**97.4**	**97.2**	**100**	86.3
20	lean	24.7	45	F	**97.9**	**97.5**	**99.5**	81.7
26	lean	24.9	35	F	**98.9**	**92.8**	**99**	82.0
16	lean	25	39	F	**95.6**	**94.9**	**99.1**	86.5
124	lean	21.6	35	F	**97.4**	**94.8**	**95.6**	77.6
129	lean	21.9	40	F	**95**	**94**	**93**	75.2
28	lean	23.9	47	F	**98.6**	94.4	90.7	81.5
30	lean	23.4	37	F	**97.1**	100	98.3	73.7
31	lean	20.3	32	F	**95**	93.5	99	85.0
27	lean	20.8	30	F	**100**	93.7	97.2	84.5
98	obese	29.8	27	F	**94.1**	96.1	97.3	65.1
116	obese	30.1	27	F	**96.7**	95.7	98.8	76.7
109	obese	30.5	31	F	**97.8**	96.4	96.2	67.5
117	obese	32.2	18	F	**97.1**	97.1	93.7	83.6
100	obese	30.6	29	F	**98.2**	94.7	98.4	71.8
57	obese	32.2	31	M	**95.3**	92.5	80.8	78.0
29	obese	32.4	52	F	**96.7**	93	97.1	73.5
144	obese	37	42	M	**95.2**	93.7	99.2	68.2
74	obese	31.2	45	F	**94**	96.5	97.2	71.4

*Samples labeled in bold were used for RNA expression profiling.

**Table 2:** Antibodies used for FACS sorting.

**Specificity**	**Clone**	**Color**	**Company**
anti-CD45	T29/33	Pacific Blue	DakoCytomation, Glostrup, Denmark
anti-CD14	M5E2	PE	BD biosciences, San Jose, CA, USA
anti-CD34	8G12	APC	BD biosciences, San Jose, CA, USA
anti-CD3	SK7	PerCP-Cy5.5	BD biosciences, San Jose, CA, USA
anti-CD31	WM59	FITC	BD biosciences, San Jose, CA, USA

**Table 3:** Sybrgreen primer sequences.

**Gene name**	**Forward primer**	**Reverse primer**
CNTN3	GAGAACTGTCATATGCTTGG	CTTAGATATGTAGAGGTGCCC
ENPEP	CTACACTCTTGAGCAATACC	ACCTTGACAAAAGAGTAACG
HAS2	GATGCATTGTGAGAGGTTTC	CCGTTTGGATAAACTGGTAG
PLIN1	CAGAATGAAGACCTAAATGACC	ATGCATCGTACCATCTACTG
18S	TGACTCAACACGGGAAACC	TCGCTCCACCAACTAAGAAC
